# Synthesis and crystal structure of *N* ^1^,*N* ^2^-di­methyl­ethane­dihydrazide

**DOI:** 10.1107/S2056989024000239

**Published:** 2024-01-12

**Authors:** Yurii S. Bibik, Dmytro M. Khomenko, Roman O. Doroshchuk, Ilona V. Raspertova, Alexandra Bargan, Rostyslav D. Lampeka

**Affiliations:** a Department of Chemistry, Kyiv National Taras Shevchenko University, Volodymyrska St 64, Kyiv, Ukraine; b Enamine Ltd., Winston Churchill St 78, Kyiv 02094, Ukraine; c "Petru Poni" Institute of Macromolecular Chemistry, Aleea Gr. Ghica Voda 41A, 700487 Iasi, Romania; Katholieke Universiteit Leuven, Belgium

**Keywords:** crystal structure, X-ray crystallography, hydrazide, hydrogen bonds

## Abstract

The title compound, *N* ^1^,*N* ^2^-di­methyl­ethane­dihydrazide, C_4_H_10_N_4_O_2_, was obtained by the methyl­ation of oxalyl dihydrazide protected with phthalimide. The mol­ecule is essentially non-planar with a dihedral angle between the two planar hydrazide fragments of 86.5 (2)°. This geometry contributes to the formation of a multi-contact three-dimensional supra­molecular network *via* C—H⋯O, N—H⋯O and N—H⋯N hydrogen bonds.

## Abstract 2

In the title compound, the two hydrazide planes make a dihedral angle of 86.5 (2)°. In the crystal, C—H⋯O, N—H⋯O and N—H⋯N hydrogen bonds lead to the formation of a three-dimensional supra­molecular network.

## 1. Chemical context

For over a century, researchers have aimed to synthesize diverse heterocycles using well-established available methods. Currently, there is significant research inter­est in developing new methods for their synthesis, focusing on efficient and atom-economical routes (Favi, 2020 ▸; Pathan *et al.*, 2020 ▸). Among these novel synthetic approaches, the utilization of hydrazides stands out as one of the most appealing methods for synthesizing heterocyclic compounds such as pyrazoles, triazoles, oxa­diazo­les and pyridazines (Majumdar *et al.*, 2014 ▸; Mittersteiner *et al.*, 2021 ▸; Hosseini & Bayat, 2018 ▸; Khomenko *et al.*, 2022 ▸).

Organic acid hydrazides constitute a broad group of hydrazine derivatives containing the functional group –C(=O)NHNH_2_. Therefore, this keen inter­est in hydrazide chemistry appears to arise not only from their diversity but also from the unique properties of these compounds. Acid hydrazides and their derivatives such as hydrazones possess biological activities including anti­convulsant (Angelova *et al.*, 2016 ▸), anti­depressant (Ergenç *et al.*, 1998 ▸), anti-inflammatory (Kajal *et al.*, 2014 ▸), anti­malarial (Walcourt *et al.*, 2004 ▸), anti­mycobacterial (Shalini *et al.*, 2019 ▸), anti­cancer (Witusik-Perkowska *et al.*, 2023 ▸; Küçükgüzel *et al.*, 2015 ▸) and anti­microbial (Hiremathad *et al.*, 2015 ▸; Popiołek *et al.*, 2022 ▸; Berillo & Dyusebaeva, 2022 ▸; Popiołek, 2021 ▸). Hydrazides are also bidentate ligands that can form chelate complexes (Ju *et al.*, 2023 ▸). 
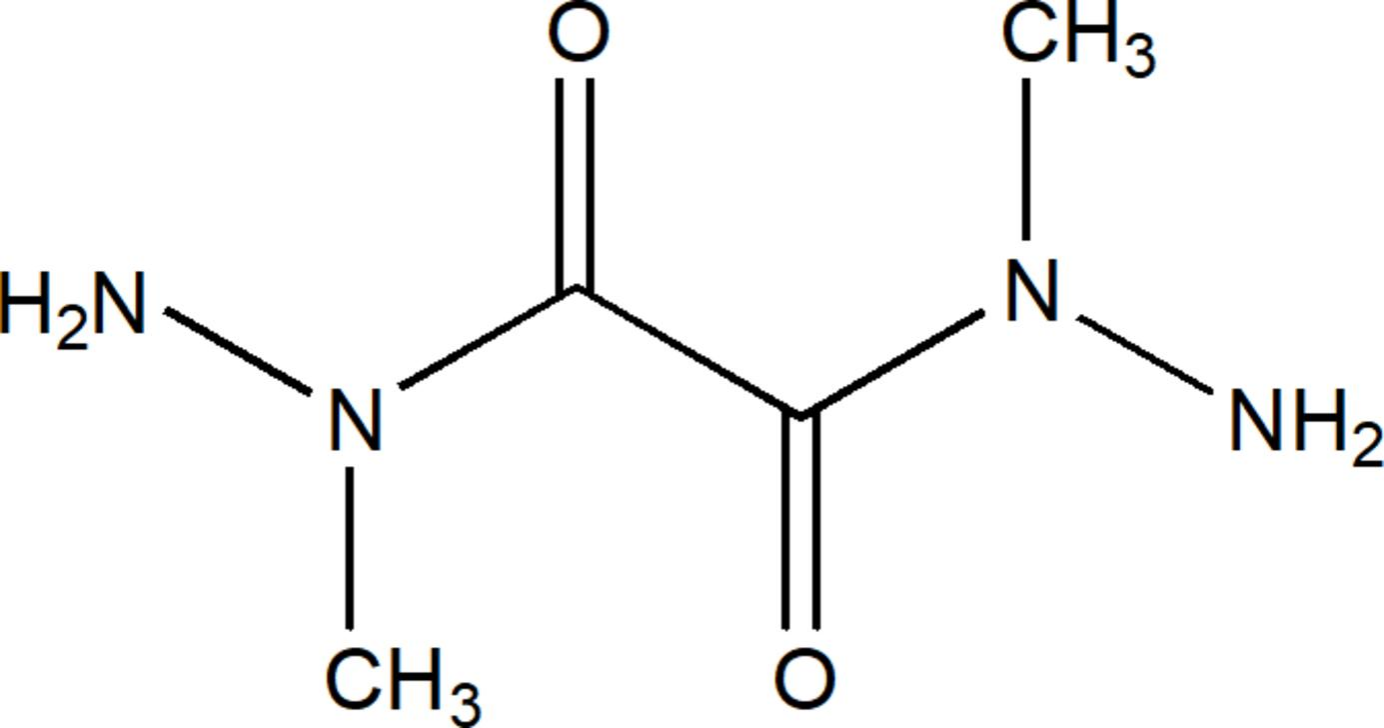


Considering the above, we report on the synthesis and crystal structure of a new alkyl­ated oxalyl dihydrazide as an attractive synthon for the synthesis of biologically active organic compounds and metal complexes.

## 2. Structural commentary

**Figure 1:**
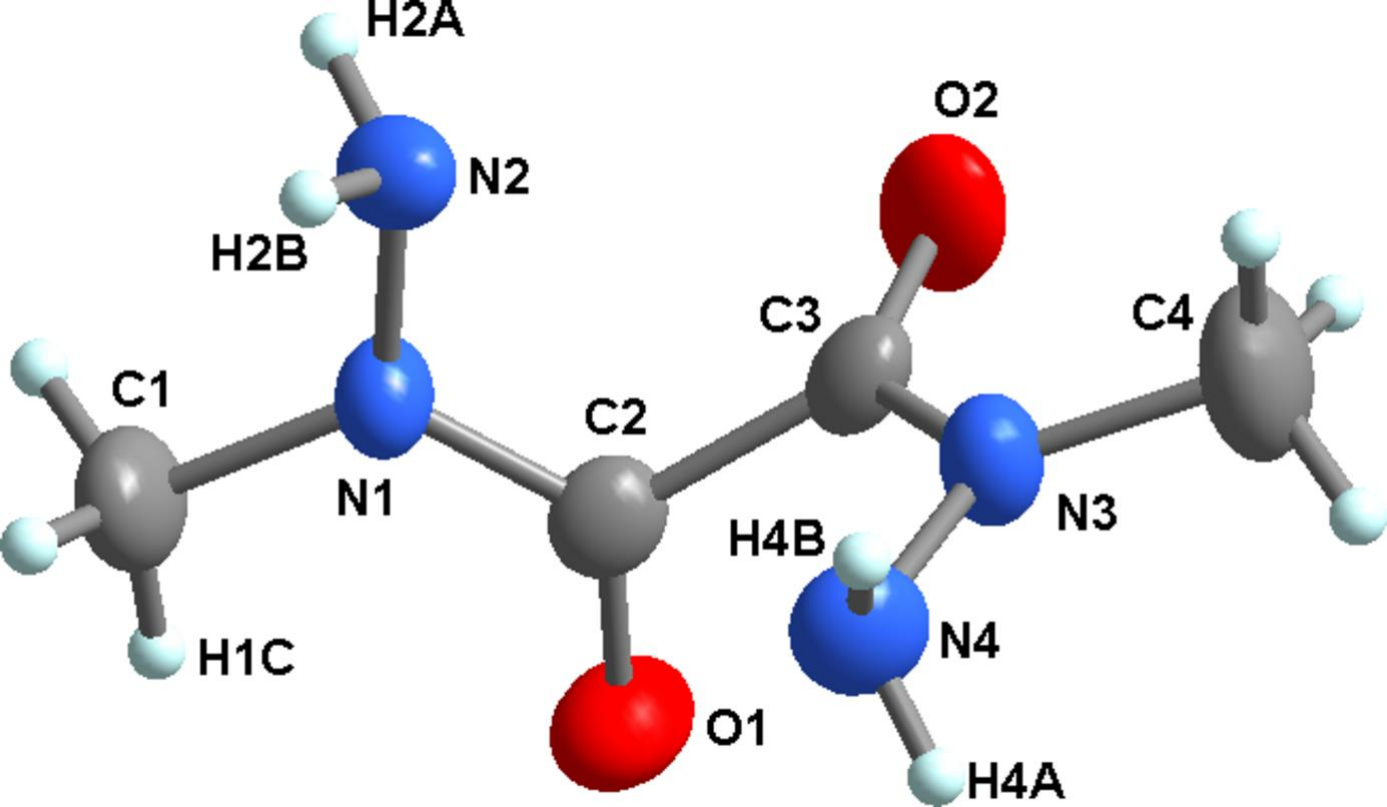
The mol­ecular structure of the title compound with atom labeling and displacement ellipsoids drawn at the 50% probability level.

The title compound crystalizes in the ortho­rhom­bic Sohncke space group *P*2_1_2_1_2_1_ with four formula units per unit cell (Fig. 1 ▸). The crystal structure does not show other tautomeric forms. Bond lengths and angles are given in Table 1 ▸. The geometrical parameters are comparable to the values found in methyl­semicarbazide (Szimhardt & Stierstorfer, 2018 ▸) and oxalyl dihydrazide (Quaeyhaegens *et al.*, 1990 ▸). The methyl hydrazide core [–C(=O)N(—CH_3_)NH_2_] is almost planar (r.m.s. deviation = 0.022 Å). The torsion angles around the N1—C2 and N3—C3 bonds are N2—N1—C2—O1 = 175.1 (4)°, C1—N1—C2—O1 = −1.2 (5)°, N4—N3—C3—O2 = 174.8 (4)°, and C4—N3—C3—O2 = −1.4 (6)°. The methyl hydrazide fragments are almost perpendicular to each other [the dihedral angle between the two moieties is 86.5 (2)°]. The torsion angles around the C2—C3 bond are O1—C2—C3—O2 = 89.9 (4), O1—C2—C3—N3 = −81.4 (4), N1—C2—C3—O2 = −83.2 (4), and N1—C2—C3—N3 = 105.5 (4)°.

## 3. Supra­molecular features

**Figure 2:**
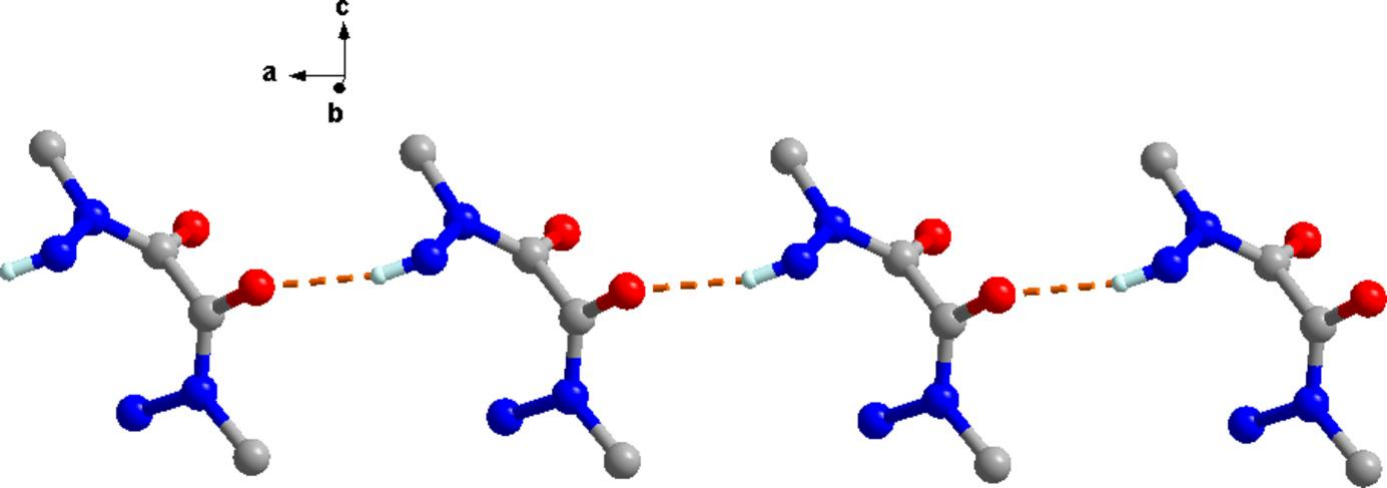
One-dimensional chains along the *a*-axis direction formed by N—H⋯O hydrogen bonding.

**Figure 3:**
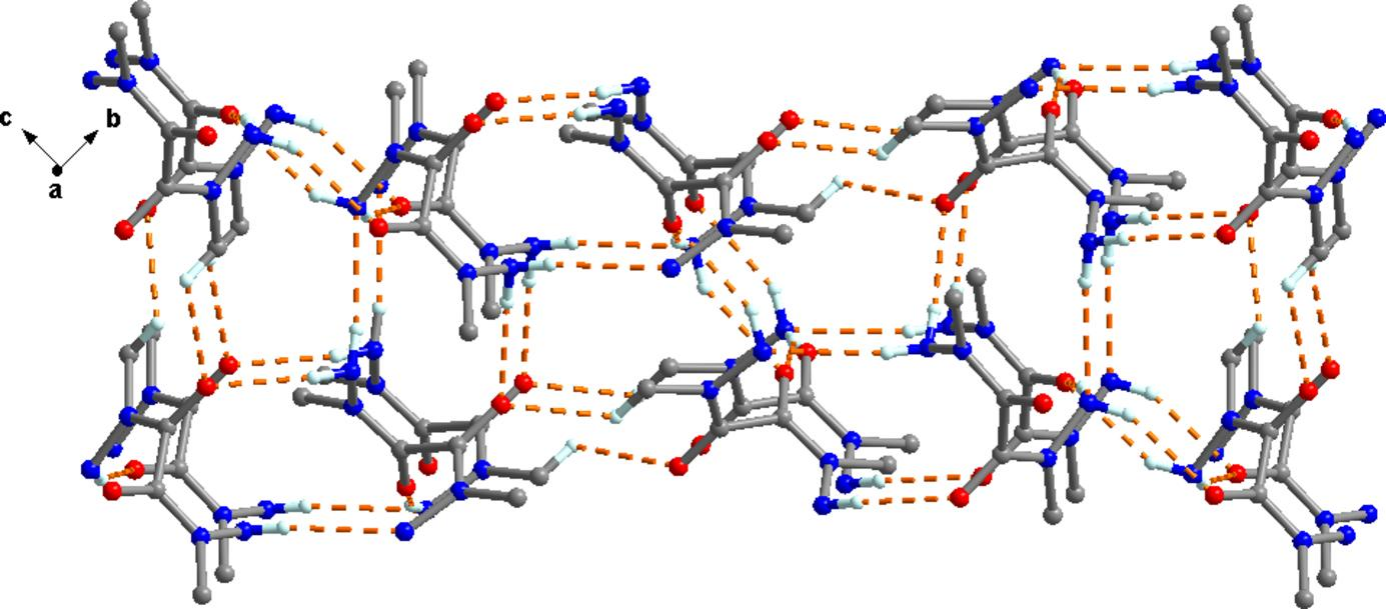
A view normal to plane (100) of the crystal structure of the title compound, showing the three-dimensional supra­molecular hydrogen-bonding network.

In the crystal, each mol­ecule forms chains along the *a*-axis direction with two neighboring ones *via* N—H⋯O hydrogen bonds (Table 2 ▸, Fig. 2 ▸). Neighboring chains form a 3D supra­molecular network *via* C—H⋯O, N—H⋯O and N—H⋯N hydrogen-bonding contacts (Table 2 ▸, Fig. 3 ▸).

## 4. Database survey

A search of the Cambridge Structural Database (CSD, version 5.43, last update November 2021; Groom *et al.*, 2016 ▸) confirmed that the title compound has not previously been published. A search for the N—N—C(=O)—C(=O)—N—N fragment gave oxalyl dihydrazide (CSD refcode VIPKIO; Quaeyhaegens *et al.*, 1990 ▸), its salts: EREQOK (Wu, 2021 ▸), NEXMIP (Xu *et al.*, 2018 ▸), MIDNOG (Devi *et al.*, 2018 ▸), VUHYUU and VUHZAB (Fischer *et al.*, 2014 ▸), ZIBBIX and ZIBDAR (Fischer *et al.*, 2013 ▸), and Schiff bases derived from it as the closest analogues: CUQPAF (Drexler *et al.*, 1999 ▸), HIRHIB (Singh *et al.*, 2013 ▸), IYACUH (Ran *et al.*, 2011 ▸), KUTREX (Kaluderović *et al.*, 2010 ▸), LORQEP (Bi *et al.*, 2009 ▸), NAJWUT (Singh *et al.*, 2016 ▸), NEQQOQ (Zhu *et al.*, 2006 ▸), RIRTET (Singh *et al.*, 2014 ▸), SUYWUG (Galvão *et al.*, 2016 ▸), UMIZUN (El-Asmy *et al.*, 2015 ▸), ZOLQUP and ZOLRAW (Fries *et al.*, 2019 ▸). For compound ZOLQOJ (Fries *et al.*, 2019 ▸), the fragment is part of a ring structure. Notably, a strictly planar structure is observed for the mol­ecules oxalyl dihydrazide VIPKIO and dimethyl oxalate DMEOXA (Dougill & Jeffrey, 1953 ▸).

A search for the methyl hydrazide moiety gave methyl­semicarbazide (XIBFEW; Szimhardt & Stierstorfer, 2018 ▸). Its geometric parameters agree well with those of the title compound. Further searches also revealed two structural analogues with a second non-hydrogen substituent at the amide-nitro­gen atom: *N*,*N*,*N*′,*N*′-tetra­methyl­oxamide and *N*,*N*,*N*′,*N*′-tetra­methyl­mono­thio­oxamide (TMOXAM and TMTHOX, respectively; Adiwidjaja & Voss, 1977 ▸). These two crystal structures have a different packing and belong to monoclinic space groups. However, they exhibit very similar geometries in terms of the rotation of the mol­ecule fragments around the central C—C bond. The O=C—C=O(S) torsion angles are 105.1 (2) and 89.6 (2)°, respectively.

## 5. Synthesis and crystallization

**Figure 4:**
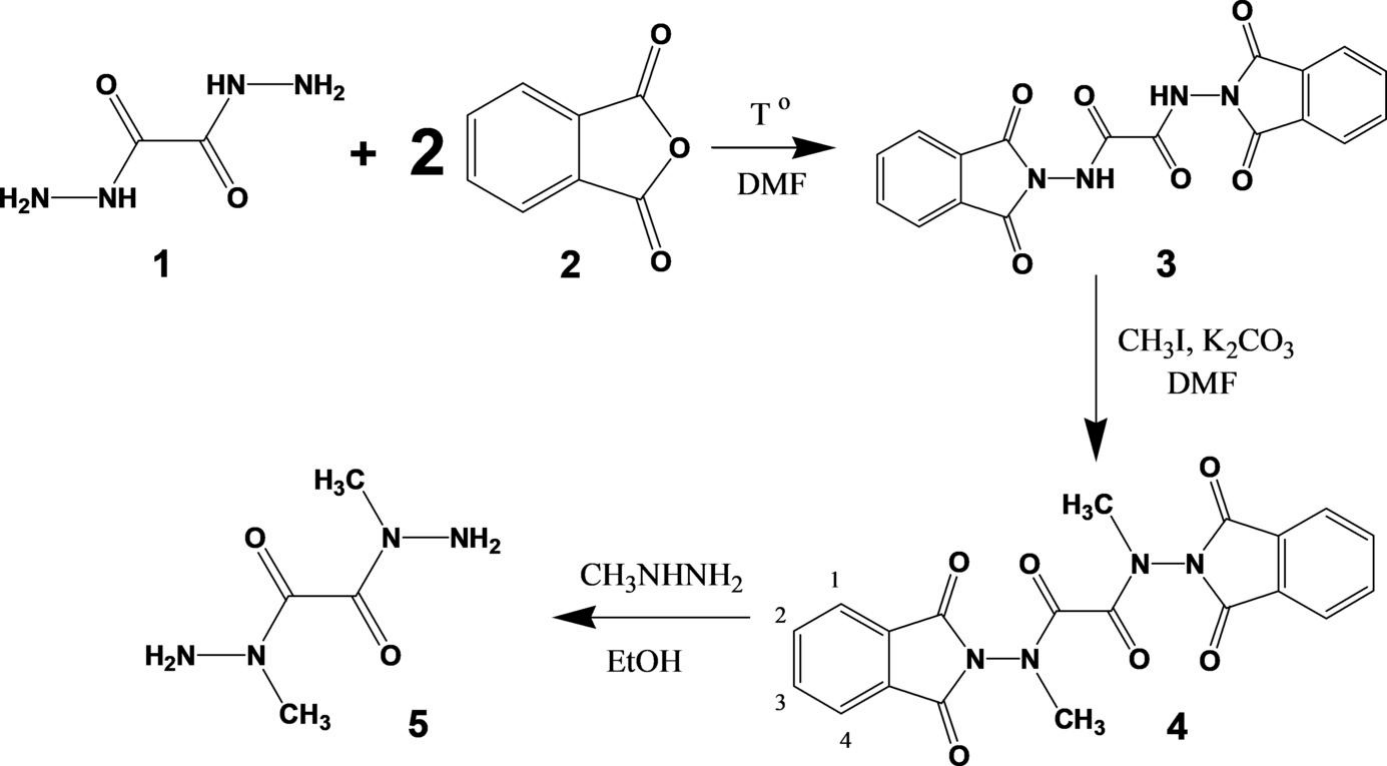
Synthesis of the title compound.

The title compound (**5**) was obtained according to the reaction scheme shown in Fig. 4 ▸.

***N***, ***N*** **’-bis­(1,3-dioxo-1,3-di­hydro-2** ***H*** **-isoindol-2-yl)ethanedi­amide (3)**: compound **3** was synthesized from the commercially available precursors (Enamine Ltd.) according to the following method: 12.45 g (84 mmol, 2 eq.) of phthalic anhydride (**2**) were dissolved in 125 ml of DMF and 4.96 g (42 mmol, 1 eq.) of oxalyl dihydrazide (**1**) were added to the boiling solution. The obtained mixture was refluxed for 5 h. Upon cooling, precipitation of the product was observed. It was filtered off and dried. White powder; yield 73%. ^1^H NMR (400 MHz, DMSO-*d*_6_): *δ* 8.05–8.15 (*m*, 4H, 4-Ph), 11.57 (*br*, 1H, NH).

***N*** **^1^**, ***N*** **^2^-di­methyl­ethane­dihydrazide (5):** 11.0 g (79.7 mmol, 3 eq.) of K_2_CO_3_ and 3.65 ml (58.6 mmol, 2.2 eq.) of CH_3_I were added to a solution containing 10.0 g (26.5 mmol, 1 eq.) of compound **3** in 50 ml DMF. The reaction mixture was stirred for 6 h at room temperature. The inorganic precipitate was filtered off, the filtrate was evaporated and the residue was stirred in water, filtered off and dried in air. Yield: 9.9 g.

The crude precipitate of **4** (4 g, 9.8 mmol, 1 eq.) obtained from the previous step was refluxed with 1.1 ml (20.6 mmol, 2.1 eq.) of methyl­hydrazine in ethanol for 6 h. The precipitate was filtered off, ethanol was evaporated and the residue was recrystallized from 2-propanol and dried in air. The title compound was isolated as a white solid. Crystals suitable for X-ray analysis were obtained during the recrystallization. White powder; yield 84%. LC–MS (ESI) *m*/*z* 147 (MH^+^) . IR (ATR, *ν*, cm^−1^) : *ν* 3290, 3214, 1672, 1616, 1414, 1386, 1234, 1066, 1014, 870, 782, 762. ^1^H NMR (400 MHz, DMSO-*d*_6_): *δ* 2.90*, 2.95 and 3.00* (*s*, 3H, CH_3_), 4.68, 4.85* and 4.93* (*s*, 2H, NH_2_). *Minor signals indicate hindered rotation about the (O)C—N bond.

## 6. Refinement

Crystal data, data collection and structure refinement details are summarized in Table 3 ▸. For the NH_2_ group, the hydrogen atoms were placed from a difference-Fourier map and refined freely. The CH_3_ hydrogen atoms were placed geometrically and refined as riding with C—H = 0.96 Å and *U*_iso_(H) = 1.5*U*_eq_(C).

## Supplementary Material

Crystal structure: contains datablock(s) I. DOI: 10.1107/S2056989024000239/vm2294sup1.cif

Structure factors: contains datablock(s) I. DOI: 10.1107/S2056989024000239/vm2294Isup4.hkl

CCDC reference: 2323887

Additional supporting information:  crystallographic information; 3D view; checkCIF report

## Figures and Tables

**Table 1:** Selected geometric parameters (Å, °)

O1—C2	1.231 (4)	N3—N4	1.414 (4)
O2—C3	1.233 (4)	N3—C3	1.319 (4)
N1—N2	1.412 (4)	N3—C4	1.460 (4)
N1—C1	1.460 (4)	C2—C3	1.511 (5)
N1—C2	1.331 (4)		
			
N2—N1—C1	119.9 (3)	O1—C2—N1	123.8 (3)
C2—N1—N2	117.6 (3)	O1—C2—C3	118.2 (3)
C2—N1—C1	122.4 (3)	N1—C2—C3	117.7 (3)
N4—N3—C4	120.5 (3)	O2—C3—N3	124.1 (4)
C3—N3—N4	117.3 (3)	O2—C3—C2	118.7 (3)
C3—N3—C4	122.1 (4)	N3—C3—C2	116.5 (3)

**Table 2:** Hydrogen-bond geometry (Å, °)

*D*—H⋯*A*	*D*—H	H⋯*A*	*D*⋯*A*	*D*—H⋯*A*
C1—H1*C*⋯O1^i^	0.96	2.58	3.042 (4)	110
N2—H2*A*⋯O2^ii^	0.87 (5)	2.13 (5)	2.977 (4)	164 (4)
N2—H2*B*⋯O2^iii^	0.90 (3)	2.35 (3)	3.182 (4)	155 (3)
N4—H4*A*⋯O1^iv^	0.79 (4)	2.30 (4)	3.075 (5)	169 (4)
N4—H4*B*⋯N2^v^	0.99 (5)	2.38 (5)	3.367 (5)	170 (4)

Symmetry codes: (i) 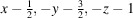 ; (ii) 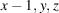 ; (iii) 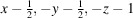 ; (iv) 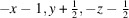 ; (v) 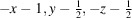 .

**Table 3:** Experimental details

Crystal data	
Chemical formula	C_4_H_10_N_4_O_2_
*M*_r_	146.16
Crystal system, space group	Orthorhombic, *P*2_1_2_1_2_1_
Temperature (K)	293
*a*, *b*, *c* (Å)	6.0356 (5), 7.6501 (6), 15.7851 (14)
*V* (Å^3^)	728.84 (10)
*Z*	4
Radiation type	Mo *K*α
μ (mm^−1^)	0.11
Crystal size (mm)	0.25 × 0.2 × 0.15

Data collection	
Diffractometer	Xcalibur, Eos
Absorption correction	Multi-scan (*CrysAlis PRO*; Rigaku OD, 2021 ▸)
*T*_min_, *T*_max_	0.975, 1.000
No. of measured, independent and observed [*I* > 2σ(*I*)] reflections	2356, 1279, 1014
*R*_int_	0.027
(sin θ/λ)_max_ (Å^−1^)	0.595

Refinement	
*R*[*F* ^2^ > 2σ(*F* ^2^)], *wR*(*F* ^2^), *S*	0.050, 0.093, 1.04
No. of reflections	1279
No. of parameters	107
No. of restraints	6
H-atom treatment	H atoms treated by a mixture of independent and constrained refinement
Δρ_max_, Δρ_min_ (e Å^−3^)	0.14, −0.12
Absolute structure	Flack *x* determined using 280 quotients [(*I* ^+^)−(*I* ^−^)]/[(*I* ^+^)+(*I* ^−^)] (Parsons et al., 2013 ▸)
Absolute structure parameter	−0.7 (10)

Computer programs: *CrysAlis PRO* (Rigaku OD, 2021 ▸), *SHELXT* (Sheldrick, 2015*a* ▸), *SHELXL2018/3* (Sheldrick, 2015*b* ▸), and *OLEX2* (Dolomanov *et al.*, 2009 ▸).

## supplementary crystallographic information

### Crystal data

**Table:**

C_4_H_10_N_4_O_2_	*D*_x_ = 1.332 Mg m^−^^3^
*M**_r_* = 146.16	Mo *K*α radiation, λ = 0.71073 Å
Orthorhombic, *P*2_1_2_1_2_1_	Cell parameters from 809 reflections
*a* = 6.0356 (5) Å	θ = 2.6–21.5°
*b* = 7.6501 (6) Å	µ = 0.11 mm^−^^1^
*c* = 15.7851 (14) Å	*T* = 293 K
*V* = 728.84 (10) Å^3^	Prism, clear light colourless
*Z* = 4	0.25 × 0.2 × 0.15 mm
*F*(000) = 312	

### Data collection

**Table:**

Xcalibur, Eos diffractometer	1279 independent reflections
Radiation source: fine-focus sealed X-ray tube, Enhance (Mo) X-ray Source	1014 reflections with *I* > 2σ(*I*)
Graphite monochromator	*R*_int_ = 0.027
Detector resolution: 8.0797 pixels mm^-1^	θ_max_ = 25.0°, θ_min_ = 2.6°
ω scans	*h* = −4→7
Absorption correction: multi-scan (CrysAlisPro; Rigaku OD, 2021)	*k* = −6→9
*T*_min_ = 0.975, *T*_max_ = 1.000	*l* = −17→18
2356 measured reflections	

### Refinement

**Table:**

Refinement on *F*^2^	Hydrogen site location: difference Fourier map
Least-squares matrix: full	H atoms treated by a mixture of independent and constrained refinement
*R*[*F*^2^ > 2σ(*F*^2^)] = 0.050	*w* = 1/[σ^2^(*F*_o_^2^) + (0.0311*P*)^2^] where *P* = (*F*_o_^2^ + 2*F*_c_^2^)/3
*wR*(*F*^2^) = 0.093	(Δ/σ)_max_ < 0.001
*S* = 1.04	Δρ_max_ = 0.14 e Å^−^^3^
1279 reflections	Δρ_min_ = −0.11 e Å^−^^3^
107 parameters	Absolute structure: Flack *x* determined using 280 quotients [(*I*^+^)-(*I*^-^)]/[(*I*^+^)+(*I*^-^)] (Parsons et al., 2013)
6 restraints	Absolute structure parameter: −0.7 (10)
Primary atom site location: dual	

### Special details

**Table:**

Geometry. All esds (except the esd in the dihedral angle between two l.s. planes) are estimated using the full covariance matrix. The cell esds are taken into account individually in the estimation of esds in distances, angles and torsion angles; correlations between esds in cell parameters are only used when they are defined by crystal symmetry. An approximate (isotropic) treatment of cell esds is used for estimating esds involving l.s. planes.

### Fractional atomic coordinates and isotropic or equivalent isotropic displacement parameters (Å^2^)

**Table:**

	*x*	*y*	*z*	*U*_iso_*/*U*_eq_	
O1	−0.3495 (5)	−0.6601 (3)	−0.39158 (16)	0.0534 (8)	
O2	−0.1053 (4)	−0.3057 (4)	−0.38063 (17)	0.0565 (8)	
N1	−0.5715 (5)	−0.4389 (3)	−0.43410 (17)	0.0356 (8)	
N2	−0.6265 (6)	−0.2617 (4)	−0.4206 (2)	0.0390 (8)	
N3	−0.2831 (5)	−0.3723 (4)	−0.2592 (2)	0.0418 (8)	
N4	−0.4758 (7)	−0.4508 (6)	−0.2252 (2)	0.0510 (10)	
C1	−0.7056 (7)	−0.5453 (5)	−0.4913 (2)	0.0520 (11)	
H1A	−0.703336	−0.494920	−0.546990	0.078*	
H1B	−0.855356	−0.549500	−0.470930	0.078*	
H1C	−0.646056	−0.661570	−0.493510	0.078*	
C2	−0.4040 (6)	−0.5050 (5)	−0.3896 (2)	0.0357 (9)	
C3	−0.2571 (6)	−0.3787 (4)	−0.3421 (2)	0.0359 (9)	
C4	−0.1283 (7)	−0.2793 (5)	−0.2043 (3)	0.0661 (13)	
H4C	−0.188175	−0.166675	−0.190369	0.099*	
H4D	0.010785	−0.264855	−0.232939	0.099*	
H4E	−0.105945	−0.345315	−0.153239	0.099*	
H2A	−0.763 (8)	−0.255 (5)	−0.404 (2)	0.061 (14)*	
H4A	−0.537 (7)	−0.378 (5)	−0.199 (2)	0.053 (15)*	
H2B	−0.621 (6)	−0.208 (5)	−0.471 (2)	0.056 (14)*	
H4B	−0.432 (9)	−0.548 (7)	−0.187 (3)	0.12 (2)*	

### Atomic displacement parameters (Å^2^)

**Table:**

	*U*^11^	*U*^22^	*U*^33^	*U*^12^	*U*^13^	*U*^23^
O1	0.0640 (19)	0.0427 (15)	0.0536 (18)	0.0182 (15)	−0.0038 (16)	−0.0062 (13)
O2	0.0318 (15)	0.084 (2)	0.0542 (18)	−0.0106 (15)	0.0054 (15)	0.0102 (15)
N1	0.0346 (17)	0.0386 (16)	0.0335 (17)	0.0029 (15)	−0.0068 (15)	−0.0064 (14)
N2	0.0321 (19)	0.0372 (18)	0.048 (2)	0.0026 (17)	0.0011 (18)	0.0011 (17)
N3	0.0361 (18)	0.051 (2)	0.0377 (19)	−0.0037 (18)	−0.0046 (16)	−0.0015 (15)
N4	0.058 (3)	0.056 (2)	0.039 (2)	−0.002 (2)	0.0052 (19)	−0.002 (2)
C1	0.060 (3)	0.055 (2)	0.041 (2)	−0.005 (2)	−0.013 (2)	−0.011 (2)
C2	0.037 (2)	0.043 (2)	0.0267 (19)	0.0058 (19)	0.0072 (19)	−0.0023 (17)
C3	0.029 (2)	0.044 (2)	0.035 (2)	0.0081 (19)	−0.0022 (18)	0.0048 (18)
C4	0.057 (3)	0.084 (3)	0.057 (3)	−0.008 (3)	−0.017 (3)	−0.015 (3)

### Geometric parameters (Å, º)

**Table:**

O1—C2	1.231 (4)	N4—H4A	0.79 (4)
O2—C3	1.233 (4)	N4—H4B	0.99 (5)
N1—N2	1.412 (4)	C1—H1A	0.9599
N1—C1	1.460 (4)	C1—H1B	0.9601
N1—C2	1.331 (4)	C1—H1C	0.9600
N2—H2A	0.87 (4)	C2—C3	1.511 (5)
N2—H2B	0.89 (4)	C4—H4C	0.9600
N3—N4	1.414 (4)	C4—H4D	0.9600
N3—C3	1.319 (4)	C4—H4E	0.9601
N3—C4	1.460 (4)		
			
N2—N1—C1	119.9 (3)	H1A—C1—H1B	109.5
C2—N1—N2	117.6 (3)	H1A—C1—H1C	109.5
C2—N1—C1	122.4 (3)	H1B—C1—H1C	109.5
N1—N2—H2A	109 (3)	O1—C2—N1	123.8 (3)
N1—N2—H2B	108 (2)	O1—C2—C3	118.2 (3)
H2A—N2—H2B	106 (4)	N1—C2—C3	117.7 (3)
N4—N3—C4	120.5 (3)	O2—C3—N3	124.1 (4)
C3—N3—N4	117.3 (3)	O2—C3—C2	118.7 (3)
C3—N3—C4	122.1 (4)	N3—C3—C2	116.5 (3)
N3—N4—H4A	107 (3)	N3—C4—H4C	109.4
N3—N4—H4B	109 (3)	N3—C4—H4D	109.6
H4A—N4—H4B	109 (4)	N3—C4—H4E	109.4
N1—C1—H1A	109.6	H4C—C4—H4D	109.5
N1—C1—H1B	109.5	H4C—C4—H4E	109.5
N1—C1—H1C	109.4	H4D—C4—H4E	109.5
			
O1—C2—C3—O2	89.9 (4)	N4—N3—C3—O2	174.8 (4)
O1—C2—C3—N3	−81.4 (4)	N4—N3—C3—C2	−14.4 (5)
N1—C2—C3—O2	−83.2 (4)	C1—N1—C2—O1	−1.2 (5)
N1—C2—C3—N3	105.5 (4)	C1—N1—C2—C3	171.6 (3)
N2—N1—C2—O1	175.1 (4)	C4—N3—C3—O2	−1.4 (6)
N2—N1—C2—C3	−12.2 (4)	C4—N3—C3—C2	169.4 (3)

### Hydrogen-bond geometry (Å, º)

**Table:**

*D*—H···*A*	*D*—H	H···*A*	*D*···*A*	*D*—H···*A*
C1—H1*C*···O1^i^	0.96	2.58	3.042 (4)	110
N2—H2*A*···O2^ii^	0.87 (5)	2.13 (5)	2.977 (4)	164 (4)
N2—H2*B*···O2^iii^	0.90 (3)	2.35 (3)	3.182 (4)	155 (3)
N4—H4*A*···O1^iv^	0.79 (4)	2.30 (4)	3.075 (5)	169 (4)
N4—H4*B*···N2^v^	0.99 (5)	2.38 (5)	3.367 (5)	170 (4)

Symmetry codes: (i) *x*−1/2, −*y*−3/2, −*z*−1; (ii) *x*−1, *y*, *z*; (iii) *x*−1/2, −*y*−1/2, −*z*−1; (iv) −*x*−1, *y*+1/2, −*z*−1/2; (v) −*x*−1, *y*−1/2, −*z*−1/2.
